# Lutzomyia umbratilis from an area south of the Negro River is refractory to in vitro interaction with Leishmania guyanensis

**DOI:** 10.1590/0074-02760170425

**Published:** 2018-03

**Authors:** Rodrigo Pedro Soares, Paula Monalisa Nogueira, Nágila Francinete Secundino, Eric Fabrício Marialva, Cláudia Maria Ríos-Velásquez, Felipe Arley Costa Pessoa

**Affiliations:** 1 Fundação Oswaldo Cruz-Fiocruz, Instituto René Rachou, Belo Horizonte, MG, Brasil; 2 Fundação Oswaldo Cruz-Fiocruz, Instituto Leônidas e Maria Deane, Manaus, AM, Brasil

**Keywords:** Lutzomyia umbratilis, Leishmania guyanensis, host-parasite interaction, American cutaneous leishmaniasis (ACL)

## Abstract

**BACKGROUND:**

*Lutzomyia umbratilis*, the vector for *Leishmania guyanensis* in northern South America, has been found naturally infected with *L. guyanensis* only in areas north of the Negro and Amazon rivers. While populations of this sand fly species are also found in areas south of these rivers, these populations have never been reported to be infected and/or transmitting *L. guyanensis*. However, no studies on the corresponding host-parasite interactions are available.

**OBJECTIVES:**

This study evaluated the interaction between *Lu. guyanensis* promastigotes and field-collected *Lu. umbratilis* sand flies from Rio Preto da Eva and Manacapuru, which are located to the north and south, respectively, of the Negro River.

**METHODS:**

Procyclic and metacyclic attachment was quantified using an *in vitro* system.

**FINDINGS:**

Low attachment of parasites to the midguts of insects collected from Manacapuru was detected. Conversely, greater binding of metacyclic parasites was observed in the midguts of insects collected from Rio Preto da Eva, and this attachment was more pronounced than that observed for procyclics (p < 0.03).

**MAIN CONCLUSIONS:**

The *Lu. umbratilis* population from an area south of the Negro River has lower *in vitro* interaction with *L. guyanensis*. The higher attachment of *L. guyanensis* to midguts of insects from Rio Preto da Eva may suggest better vector competence. These findings are in accordance with previously reported epidemiological information of American cutaneous leishmaniasis (ACL) transmission in the Amazon.

The sand fly *Lutzomyia* (*Nyssomyia*) *umbratilis* Ward & Frahia is the main vector of *Leishmania* (*Viannia*) *guyanensis*, an infectious agent of American cutaneous leishmaniasis (ACL) in Northern South America (Ready et al. 1985). In addition to its distribution in the Amazonian basin (Young & Duncan 1994), this species has also been detected in Atlantic Rainforest remnants in Northeast Brazil (Balbino et al. 2001); however, its local role as a transmission agent of ACL in Northeast Brazil is yet to be determined (Freitas et al. 2017).

*Lu. umbratilis* has been found to be naturally infected with *L. guyanensis* only in areas east or north of the Negro River (Arias & Freitas 1977, 1978). Arias and Freitas (1978) suggested that this river could act as a vicariant barrier to *L. guyanensis* transmission. Justiniano et al. (2004) compared the first laboratory bred generations of *Lu. umbratilis* populations obtained from areas south and north of the Negro River. Those populations exhibited remarkable biological differences in their life cycle, fecundity, fertility, and adult longevity. The northern population was more productive and lived longer compared to the southern population. These differences could be due to intrinsic biological features resulting from their geographical isolation by the Negro River. Later, Scarpassa and Alencar (2012) compared *Lu. umbratilis* populations from Manacapuru (Man; south of the Negro River) and Rio Preto da Eva (RPE; north of the Negro River) by using the cytochrome oxidase I (COI) gene and suggested that, based on its clustering, the Man population could be undergoing speciation. More recently, Freitas et al. (2015, 2016) compared these populations and included *Lu. umbratilis* specimens from Recife (in Northeast Brazil). Consistent with previous observations, two distinct clades developed: one including *Lu. umbratilis* populations from RPE and Recife and another including only the *Lu. umbratilis* population from Man.

A distinguishing feature in the epidemiology of ACL in the Manaus region is the complete absence of *L. guyanensis* transmission by *Lu. umbratilis* in Man, a city located south of the Negro River, where neither infected insects were captured nor were ACL patients found. On the other hand, in RPE, which is located north of the Negro River, cases of infection in humans often occur and infected insects are found. To understand if these distinct *Lu. umbratilis* populations have different abilities to interact with *L. guyanensis*, *in vitro* binding experiments were performed (Pimenta et al. 1992). This system allows a rapid quantitative analysis of the interaction between insect midguts and promastigote forms of *Leishmania*.

*Cell culture* - The World Health Organization reference strain of *L. guyanensis* (MHOM/BR/75/M4147) was used. This strain was isolated by Dr Ralph Lainson from a human case in Monte Dourado, northern Pará state, Brazil (Lainson et al. 1981). Promastigotes (unknown passage) were cultured in M199 medium supplemented with 10% heat inactivated foetal bovine serum (FBS), 100 units/mL penicillin, 50 μg/mL streptomycin, 12.5 mM glutamine, 0.1 M adenine, 0.0005% hemin, and 40 mM HEPES, pH 7.4, at 26°C until they reached stationary phase (Soares et al. 2002).

*Purification of peanut agglutinin (PNA)-negative cells* - Parasites from the stationary phase (1.0 x 10^7^ cells/mL) were harvested and resuspended in M199 containing PNA (*Arachis hypogaea*) at a final concentration of 35 mg/mL. This lectin has been successfully used for purification of metacyclic promastigotes in this species (Muskus et al. 1997). After a 30-min incubation at room temperature, the agglutinated parasites (PNA+) were removed by low-speed centrifugation (150 × *g*, 5 min), and metacyclic cells remaining in the supernatant (PNA) were washed twice with phosphate-buffered saline (PBS) at 2100 × *g* for 15 min at 4°C (Soares et al. 2005).

*Midgut binding studies* - Field-collected *Lu. umbratilis* sand flies from RPE (north of the Negro River, Amazonas; 2°50'50”S/59°56'28”W) and Man (south of the Negro River; 3°12'41”S/60°26'20) were used in the experiments. Both municipalities are in the northern Amazonas state, Brazil (Fig. 1). Insects were collected from tree trunk crevices by using CDC traps between 10:00 am and 12:00 pm. Taxonomic identification was performed prior to dissecting the midguts according to Young and Duncan (1994). Two consecutive binding experiments (July 2015 and August 2015) were performed in the Laboratory of Transmissible Diseases Ecology in the Amazon (ILMD/FIOCRUZ) in Manaus, Amazonas state, Brazil. In the first experiment (July 2015), 11 insects per group (PNA+ and PNA-) were dissected for each locality (Man and RPE), totaling 44 insects. In the second experiment (August 2015), 11 insects per group (PNA+ and PNA-) were dissected for each locality (Man and RPE), totaling 44 insects. Promastigote binding was quantified by an *in vitro* technique (Pimenta et al. 1994). Blood-unfed females maintained on 30% sucrose were dissected in PBS. Midguts were opened along the length of the abdominal segment by using a fine needle, placed in the concave wells of a microscope chamber slide, and individually incubated for 20 min with procyclic (PNA+) and metacyclic (PNA-) promastigotes (2 × 10^7^ cells/mL in a volume of 50 μL). Then, guts were washed in successive drops of PBS, and the number of attached parasites was determined using a Neubauer-counting chamber.

**Fig. 1:**
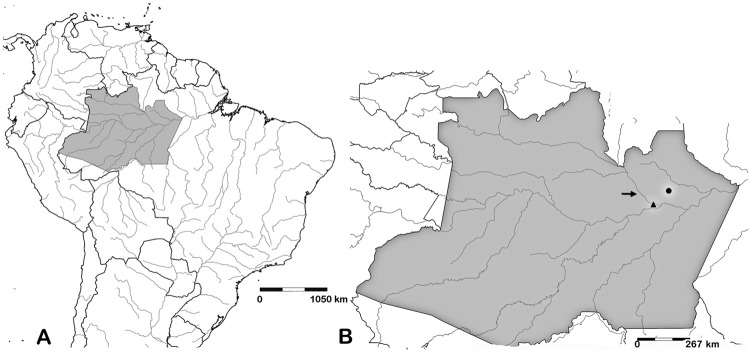
geographic location of *Lutzomyia umbratilis* populations. (A) Map of South America showing the Amazonas state, Brazil (grey); (B) collection areas in Amazonas: Rio Preto da Eva (dark circle) and Manacapuru (dark triangle). The Negro River as geographical barrier (arrow).

*Data analysis* - The Shapiro-Wilk test was employed to test the null hypothesis that data were sampled from a Gaussian distribution, where p values < 0.05 would indicate deviation from a Gaussian distribution. In such cases, a non-parametric Kruskal-Wallis test was performed, followed by Dunn's test. The Mann-Whitney test was used to compare both experiments (July and August 2015); as there were no significant differences between the two experiments (p > 0.05; Table), data were grouped (Fig. 2) and analysed using GraphPad Prism version 5.00 for Windows. (GraphPad Software, La Jolla, CA, USA; www.graphpad. com). P < 0.05 was considered statistically significant.

**Fig. 2:**
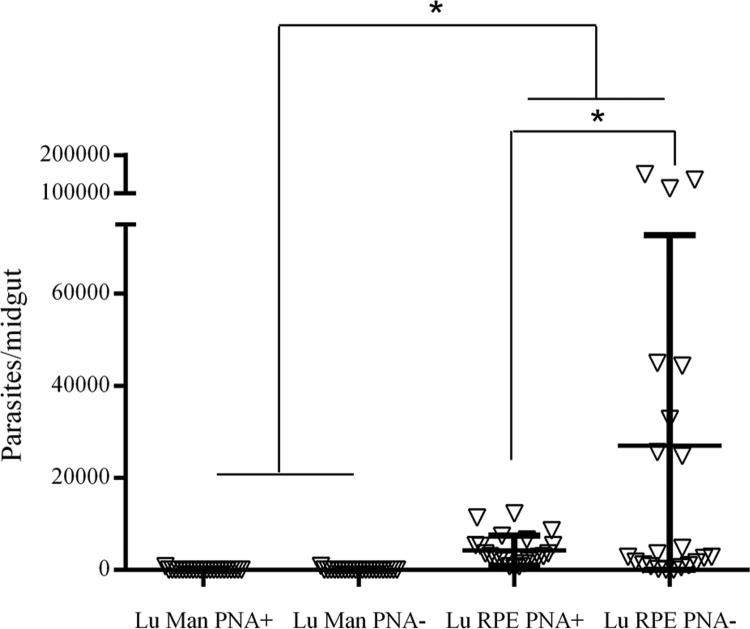
attachment of procyclic (PNA+) and metacyclic (PNA-) *Leishmania guyanensis* promastigotes to *Lutzomyia umbratilis* (Lu) mid-guts. Man: Manacapuru; RPE: Rio Preto da Eva. Data represents two independent experiments. Asterisks (*) indicate statistically significant differences (p < 0.03).

**TABLE:** *In vitro* attachment of *Leishmania guyanensis* to the midgut of *Lutzomyia umbratilis* collected from two different locations

	July 2015	August 2015	
	median (Q1, Q3)	median (Q1, Q3)	p value
Man PNA+ x Man PNA+	0.00 (0.00, 0.00)	0.00 (0.00, 0.00)	> 0.99
Man PNA- x Man PNA-	0.00 (0.00, 0.00)	0.00 (0.00, 0.00)	> 0.99
RPE PNA+ x RPE PNA+	5,195.00 (2995.00, 6,593.00)	2,398.00 (1,399.00, 2,997.00)	0.08
RPE PNA- x RPE PNA-	1,798.00 (599.40, 4,795.00)	24,726.00 (1,199.00, 44,356.00)	0.30

RPE: Rio Preto da Eva; Man: Manacapuru; PNA+: procyclic; PNA-: metacyclic; Q1: 25th percentile; Q3: (75th percentile). p < 0.05 was considered statistically significant.

Attachment to the sand fly midgut is a crucial step for *Leishmania* survival in the vector, and this is one of the interactions that determines vector competence. In Old World species such as *Phlebotomus papatasi* and *Leishmania major*, binding to the PpGalec receptor is mediated by lipophosphoglycan (LPG), a major *Leishmania* surface glycoconjugate (Kamhawi et al. 2004). However, a receptor for parasite attachment in New World species of sand flies is yet to be determined, although a midgut mucin in *Lu. longipalpis* was suggested (Myskova et al. 2016).

*In vitro* and *in vivo* protocols are available to determine parasite binding and survival inside the vector, respectively. *In vitro* protocols were developed in the early 90s to evaluate the role of phosphoglycans (PGs) in the Old World vectors *Ph. papatasi* and *Phlebotomus argentipes* (Pimenta et al. 1992, 1994). Later, the *in vitro* system was expanded to the New World species/strains *L. infantum* and *Lu. longipalpis* (Soares et al. 2002, 2017); *L. braziliensis*, *Lu. whitmani*, and *Lu. intermedia* (Soares et al. 2010, 2017); and *L. amazonensis*, *Lu. flaviscutellata, Lu. longipalpis*, and *Lu. antunesi* (de Oliveira et al. 2016).

Here, we used this *in vitro* protocol to determine the vector-parasite interaction in two *Lu. umbratilis* populations in endemic (RPE, AM) and non-endemic areas (Man, AM) for ACL. The *in vitro* protocol was selected for this study because, under the current conditions, *Lu. umbratilis* is not colonised to provide a productive off-spring (Justiniano et al. 2004). Besides, females captured from the wild refuse to feed using the artificial feeding device (FA Pessoa, unpublished data, ILMD/FIOCRUZ). After incubation of procyclic (PNA+) and metacyclic (PNA-) *L. guyanensis* promastigotes with RPE and AM sand fly populations, binding to the midgut was higher in insects from RPE in both experiments (p < 0.01). Low parasite attachment was observed in the midguts of *Lu. umbratilis* captured in Man. A unique feature of *Lu. umbratilis* binding was observed in RPE sandflies where metacyclic parasites bound to the midguts 11-fold more often than the procyclic ones (Table, Fig. 2). *L. guyanensis* is a perypilarian species from the subgenus *Viannia*, which develops in the pyloric triangle before migration to the midgut and foregut (Lainson & Shaw 1987). Previous studies showed that procyclic and metacyclic promastigotes from *L. braziliensis*, another *Viannia* subgenus species, could bind to the midgut of *Lu. whitmani* and *Lu. intermedia* (Soares et al. 2010). This is different from the subgenus *Leishmania*, where only procyclic parasites and/or PGs successfully bind to the epithelium (Pimenta et al. 1994, Mahoney et al. 1999, Kamhawi et al. 2000, Soares et al. 2002). The ability of *L. braziliensis* promastigotes to bind to the sand fly midgut may vary depending on the vector. Procyclic bind more than metacyclic promastigotes to the *Lu. whitmani* midgut, whereas in *Lu. intermedia*, they bound equally (Soares et al. 2010). It was hypothesized, based on higher attachment of *L. braziliensis* PGs, that in the species of the subgenus *Viannia*, a “second metacyclic stage” could be responsible for detachment from the midgut and further migration to the foregut (Kamhawi 2006, Assis et al. 2012). Similar to the observations in *L. braziliensis* metacyclic promastigotes, this is the first demonstration that *L. guyanensis* metacyclic promastigotes could bind to *Lu. umbratilis* midguts. However, the demonstration of a “second meta-cyclic stage” in this species is yet to be determined using *in vivo* experiments. Although the *in vitro* system has limitations (Wilson et al. 2010), it was proven to be very successful for studies on natural vector-parasite pairs.

The complete lack of interaction between *L. guyanensis* and the midgut surface of *Lu. umbratilis* collected from Man may reflect the genetic and morphometric differences in this population (Scarpassa & Alencar 2012, Freitas et al. 2015, 2016).

In conclusion, the findings herein provide the first evidence that *Lu. umbratilis* from south of the Negro River is extremely refractory to interaction with *L. guyanensis*, which supports previous epidemiological data indicating no ACL transmission due to this species in this area. According to the Killick-Kendrick (1999), *Lu. umbratilis* is a proven vector of *L. guyanensis* because it fulfills all five requirements for vectorial capacity. Furthermore, its vectorial capacity relies on many ecological factors in endemic areas corresponding to not only to their natural infection but also to their ability to transmit the parasite to humans (Lainson et al. 1981). However, the lack of *in vitro* interaction between *L. guyanensis* and insects from the Man population captured in non-endemic areas may suggest low vector competence of these insects, leading to lower vectorial capacity. Conversely, under the current experimental conditions of the *in vitro* system, specimens from the northern population (those from RPE) exhibit higher vector competence, which may reflect their improved vectorial ability for transmitting ACL in RPE.
